# The Immunomodulatory Properties of Mesenchymal Stem Cells Play a Critical Role in Inducing Immune Tolerance after Liver Transplantation

**DOI:** 10.1155/2021/6930263

**Published:** 2021-09-04

**Authors:** Shao-wei Li, Yue Cai, Xin-li Mao, Sai-qin He, Ya-hong Chen, Ling-ling Yan, Jing-jing Zhou, Ya-qi Song, Li-ping Ye, Xian-bin Zhou

**Affiliations:** ^1^Key Laboratory of Minimally Invasive Techniques & Rapid Rehabilitation of Digestive System Tumor of Zhejiang Province, Taizhou Hospital Affiliated to Wenzhou Medical University, Linhai, Zhejiang, China; ^2^Department of Gastroenterology, Taizhou Hospital of Zhejiang Province Affiliated to Wenzhou Medical University, Linhai, Zhejiang, China; ^3^Institute of Digestive Disease, Taizhou Hospital of Zhejiang Province Affiliated to Wenzhou Medical University, Linhai, Zhejiang, China; ^4^Health Management Center, Taizhou Hospital of Zhejiang Province Affiliated to Wenzhou Medical University, Linhai, Zhejiang, China; ^5^Taizhou Hospital, Zhejiang University, Linhai, Zhejiang, China

## Abstract

Although liver transplantation is considered to be the best choice for patients with end-stage liver diseases, postoperative immune rejection still cannot be overlooked. Patients with liver transplantation have to take immunosuppressive drugs for a long time or even their entire lives, in which heavy economic burden and side effects caused by the drugs have become the major impediment for liver transplantation. There is a growing body of evidences indicating that mesenchymal stem cell (MSC) transplantation, a promising tool in regenerative medicine, can be used as an effective way to induce immune tolerance after liver transplantation based on their huge expansion potential and unique immunomodulatory properties. MSCs have been reported to inhibit innate immunity and adaptive immunity to induce a tolerogenic microenvironment. In in vitro studies, transplanted MSCs show plasticity in immune regulation by altering their viability, migration, differentiation, and secretion in the interactions with the surrounding host microenvironment. In this review, we aim to provide an overview of the current understanding of immunomodulatory properties of MSCs in liver transplantation, to elucidate the potential mechanisms behind MSCs regulating immune response, especially in vivo and the influence of the microenvironment, and ultimately to discuss the feasible strategies to improve the clinical prognosis of liver transplantation. Only after exhaustive understanding of potential mechanisms of the MSC immunomodulation can we improve the safety and effectiveness of MSC treatment and achieve better therapeutic effects.

## 1. Introduction

As the most important detoxification organ, the liver is supplied by dual blood supply and may eventually develop into end-stage disease, such as decompensated liver cirrhosis, liver failure, or hepatocellular carcinoma after long-term exposure to a variety of intestinal toxins, metabolic products, or exogenous pathogens [1]. Currently, liver transplantation has been considered to be the only effective treatment for patients with end-stage liver diseases. As far back as 1963, the first case of liver transplantation was performed by Dr. Thomas Starzl for irreversible liver injury, but it did not gain popularity immediately as the discouraging results showed that no patient survived more than 23 days in the first five transplantations [2]. Until 1967, stimulated by Calne to use antilymphocyte serum, Starzl et al. began a series of successful liver transplantations [3]. However, patients with liver transplantation have to take immunosuppressive drugs for a long time or even their entire lives, in which heavy economic burden and side effects caused by the drugs (inevitable viral recurrence, metabolic complications, opportunistic infections, etc.) have become the major impediment for liver transplantation [4]. And acute graft-versus-host disease (GVHD) induced by the interaction of the innate and adaptive immune systems is also a hard nut to crack. Thus, treatments that target immune cells may be an alternative treatment to protect against severe rejection [5].

Mesenchymal stem cells (MSCs), a subpopulation of multipotent nonhematopoietic stem cells derived from neural crest mesoderm and first reported by Friedenstein et al. in 1970, are being actively studied owning to their great potential in tissue repair and immunomodulation [6, 7]. Although there are no large-scale clinical practices involving MSCs for liver transplantation and most investigations on MSCs remain in the preclinical stage, the unique immunomodulatory properties of MSCs shown in recent studies make MSC transplantation a promising tool in regenerative medicine to induce immune tolerance to various immune-related diseases [8–11]. In vitro studies, transplanted MSCs have shown plasticity in immune regulation by regulating their viability, migration, differentiation, and secretion in the interactions with the surrounding host microenvironment [12, 13]; however, the exact mechanism, especially in vivo, has yet to be fully seen.

In this review, we aim to provide an overview of the current understanding of immunomodulatory properties of MSCs in liver transplantation, to elucidate the potential mechanisms behind MSCs regulating immune response, especially in vivo and the influence of the microenvironment, and ultimately to discuss the feasible strategies to improve the clinical prognosis of liver transplantation. Meanwhile, we highlight the importance of pretreatment with cytokines, genetic modification, or three-dimensional (3D) culture in MSC-based therapy in liver transplantation. Only after exhaustive understanding of potential mechanisms of the MSC immunomodulation can we improve the safety and effectiveness of MSC treatment and achieve better therapeutic effects.

## 2. The Biological Characteristics and Research Status of MSCs

According to the International Society for Cellular Therapy (ISCT) committee, the definition of MSCs is as follows: MSCs, a subpopulation of multipotent nonhematopoietic stem cells derived from neural crest mesoderm, can differentiate into adipocytes, myocardial cells, bone cells, and chondrocytes in vitro, they are plastically adherent and fibroblast-like after culture in vitro, and they highly express cell surface markers such as CD73, CD105, and CD90 but hardly express cell surface markers such as CD14, CD34, CD45, and human leukocyte antigen- (HLA-) DR by flow cytometry [14]. Although a unique and definitive marker has not yet been found for MSCs, it is well known that they have lower expression of major histocompatibility complex- (MHC-) II and costimulatory molecules; based on this characteristic, it may partially explain why MSCs are immune privileged in vivo [15]. And as for cell therapy for immune rejection after organ transplantation, it is characterized mainly by its powerful immune regulation function.

In addition to MSCs, immunomodulatory properties appear to be a shared feature in other stromal cells including fibroblasts, hematopoietic stem cells (HSCs), and bone marrow mononuclear cells (BMCs). The infusion of autologous BMCs intravenously has been reported to significantly improve serum albumin levels and Child-Pugh score in patients with cirrhosis, and the expression levels of alpha-fetoprotein and proliferating cell nuclear antigen (PCNA) in liver biopsy tissue were significantly elevated [16]. Nevertheless, the special subpopulations of BMCs responsible for these improvements have not been determined so far. Compared to the BMCs, recent studies show that MSCs have the following distinctive advantages: MSCs can be isolated from diverse tissues [17], MSCs are easy to cultivate, expand, and store in vitro without losing clinical applicability [18], and allogeneic MSCs can be used directly without combining immunosuppressive agents [19, 20]. The potential to transdifferentiate into hepatocyte-like cells is the key for MSCs to treat liver diseases [21], but hepatocytes differentiated from MSCs in animal models were rarely more than 1% of the total liver mass [22]. Eom et al. [23] indicated that the abilities to migrate into damaged sites in a targeted manner, secrete trophic factors, and exert antifibrotic, antioxidant, and immunosuppressive effects were regarded as the therapeutic basis of MSCs in regenerative medicine.

Bone marrow (BM), as the first tissue to isolate MSCs, is still the main source of MSCs in the current clinical studies of organ transplantation. With the successful application of MSCs in many fields, its demand is gradually expanding and researchers are forced to find alternative sources of MSCs. Indeed, MSCs can be isolated not only from multiple human tissues, such as peripheral blood, umbilical cord (UC), and adipose tissue, but also from some other species including mice, rats, and rabbits. Nonetheless, care must be taken that the properties of MSCs isolated from different tissues are not completely identical, which may secrete different cytokines and exhibit different immunosuppressive effects. Ock et al. [24] confirmed that BM-MSCs showed higher self-renewal ability with a relatively slow rate in proliferation, while adipose-derived MSCs (A-MSCs) exhibit higher proliferation speed. And in this article, transforming growth factor- (TGF-) *β* and interleukin- (IL-) 10 were detected in MSCs derived from adipose and BM, respectively, whereas skin-derived MSCs simultaneously expressed both interferon- (IFN-) *γ* and IL-10. Coincidentally, Rafat et al. [25] found that expression levels of osteocalcin in BM-MSCs before and after being preconditioned with the same dose of melatonin were higher than those in A-MSCs, which also revealed the discrepancy among MSCs from diverse tissues. In addition, the function of MSCs from the same source may also be different. A recent study conducted by Semenova et al. [26] demonstrated that biological properties differed in UC-MSCs from three different regions, in which UC-MSCs derived from Wharton's jelly had higher and more stable proliferation potential and phenotype compared to those derived from the perivascular space and umbilical membrane. Another study showed that UC-MSCs in late passages produced more IL-6 and subsequently displayed stronger immunosuppressive activities than those in the early passage [27]. In view of the above research findings, the medical regimens based on MSCs must be personalized according to the patient's situation in the future, though a lot of research is still needed to achieve this goal.

More significantly, MSC not only can be isolated from above-mentioned adult tissues (BM, fat tissues, cord tissue, etc.) but also can be derived from pluripotent stem cells (PSCs) as alternative cell resources. MSCs derived from the same parental pluripotent stem cells can avoid many shortcomings of using adult MSCs, such as high variation of cell quality among batch-to-batch or donor-to-donor, stem cell senescence, and limited proliferation potential [28, 29]. As early as 2012, human PSC-derived MSCs was confirmed to possess higher proliferative capacity and stronger immunomodulation compared to BM-MSCs. Specifically, the rate of cell proliferation of human PSC-derived MSCs was demonstrated to be regulated via an ether-à-go-go 1 (hEAG1) potassium channel [30]. Most recently, a phase I, multicenter, and open-label clinical trial from the UK published in *Nat Med* [31] revealed that the application of induced pluripotent stem cell- (iPSC-) derived MSCs in the treatment of steroid-resistant acute graft-versus-host disease (SR-aGvHD) was effective and safe. In the result of this study, no serious adverse events were recorded as related to iPSC-derived MSCs, and overall response, complete response, and overall survival rates by day 100 were 86.7, 53.3, and 86.7%, respectively [31]. Of note, Qi et al. constructed a rat orthotopic liver transplantation model and indicated that genetically engineered iPSC-MSC delivering glutathione peroxidase 3 (GPx3) can effectively ameliorate inflammation and severe liver graft injury via suppressing hepatic senescence in a dose-dependent manner [32]. Collectively, the therapeutic application of PSC-derived MSCs might be promising in diverse immune-mediated diseases and provide another putative cellular source that can overcome many limitations of adult MSC.

### 2.1. The Clinical Application of MSCs for the Treatment of Liver Diseases

As early as 2004, MSCs were first reported to be used as an immunomodulator in a patient with severe treatment-resistant grade IV GVHD of the liver and provided a striking clinical response that the patient recovered well after 1 year [33]. These results not only indicated that MSCs might be a promising alternative in the treatment of GVHD even after the failure of immunosuppressive drugs but also suggested the possibility of MSCs applied in solid organ transplantation. To date, MSC application in the clinic has been reported in several studies, and there are more than 16 commercially approved MSC-based products used to treat various diseases worldwide [34]. However, large-scale clinical trials on liver disease involving MSCs are still insufficient, and none of those commercial products have been approved for the treatment of liver disease. The reasons for this phenomenon may partly lie in several internal constraints and external obstacles, such as ethical problems, source of MSCs, immune environment issues, safety and validity concerns, and MSC infusion (timing, approach, and dosage) [34, 35].

In order to conquer the many constraints and obstacles, the research concerning clinical application of MSC in liver disease has not been stopped. The majority of liver diseases using MSCs have been reported to be targeted end-stage liver diseases, including decompensated liver cirrhosis, liver failure, and complications after liver transplantation [36]. The MiSOT-I clinical trial, performed by the Mesenchymal Stem Cells in Solid Organ Transplantation (MiSOT) organization and initiated in 2013 [37], has summarized much feedback information combined with other clinical trials of liver transplantation, which included mainly the following aspects. (1) The timing of MSC infusion: MSCs need to be used early after transplantation because they have long-term tolerance-promoting effects [38]. (2) The approach of MSC infusion: a peripheral vein is the main infusion route of MSC, followed by the hepatic artery and portal vein [39].(3) The dosage of MSC infusion: the infusion dose of MSCs needs to be converted according to the weight of recipients, approximately 0.1–7 × 10^6^/kg [35, 36].(4) Safety concerns: there is no evidence showing that MSCs can increase the risk of cancerous degeneration and opportunistic infections in recent studies [40–43]. Importantly, Reinders et al. [44] conducted a clinical trial to assess the safety of allogeneic BM-derived MSC therapy in renal transplant and found that up to three patients had opportunistic but mild viral infections among the six patients injected with MSCs. Thus, given the small sample size and short follow-up period, the possibility of infections after MSCs remains to be taken seriously. (5) Validity concerns: a large number of in vivo and in vitro experiments have proven that MSCs were effective in treatments of alloimmune response after liver transplantation [41–43, 45]. A randomized controlled clinical trial by Shi et al. was conducted to evaluate the clinical feasibility of UC-MSC therapy in liver transplant patients with acute graft rejection [46]. Shi et al. found that UC-MSC could better improve liver function maybe via increasing the level of TGF-*β*1 and prostaglandin E2 and regulating the proportion of T lymphocyte subsets, compared to the control group using immunosuppressive drugs alone. Another clinical trial involving ischemic-type biliary lesions after liver transplantation showed that UC-MSC were able to improve liver function, reduce the need for interventional therapies, and increase the graft survival rate [41]. On the contrary, a phase I-II, open-label, clinical study published in *J Hepatol* in 2017 [40] indicated different findings that MSC infusion was unable to induce immune tolerance after liver transplantation and it was not successful to achieve immunosuppression weaning in MSC recipients; there was no significant difference in overall rates of rejection and graft survival, peripheral blood lymphocyte typing, and 6-onth graft biopsies between two groups, and this result might be owning to small sample size (*n* = 10), the tacrolimus-based immunosuppressive regimen, or insufficient dosage of MSCs.

Collectively, MSC therapy is mostly applied in the treatment of end-stage liver diseases, especially in the aspect of MSCs inducing immune tolerance after liver transplantation. Although the available information concerning the clinical application of MSC in liver transplantation is promising, large-scale clinical trials are still needed to further evaluate the safety and validity of MSC treatment in the near future.

### 2.2. The Potential Mechanisms of MSCs in Improving the Prognosis of Liver Transplantation

**Figure 1:**
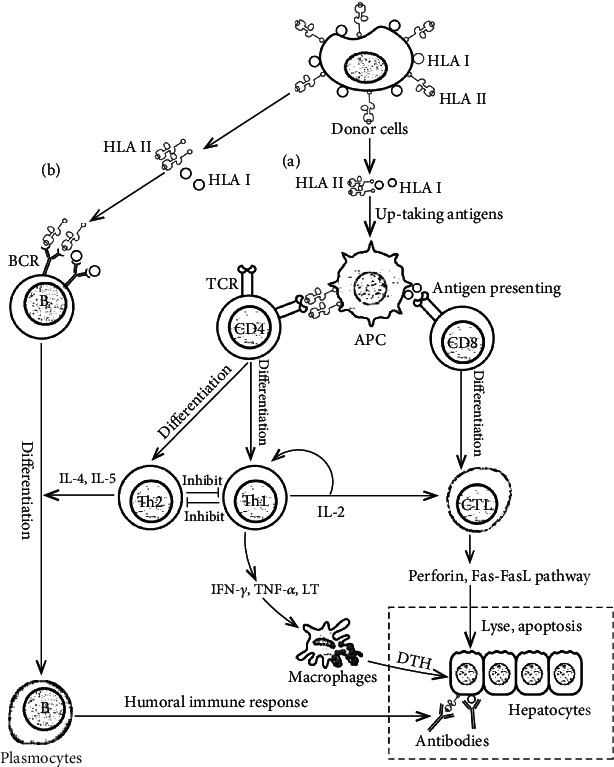
The mechanism of immune rejection after liver transplantation. (a) Immune rejection mediated by cellular immunity. Antigen-presenting cells (APCs) recognize and uptake the foreign antigens from donor-derived cells and present them to T cells, in which CD4+ T cells are activated and differentiate into various effector T cells, such as T helper 1 (Th1), Th2, and cytotoxic T lymphocytes (CTLs). Th1 cells can induce CD8+ T cells to differentiate into cytotoxic T lymphocytes (CTLs) via secreting IL2, and CTLs can secrete perforin to directly lyse hepatocytes or induce target cell apoptosis through the Fas-FasL pathway. On the other hand, Th1 cells can secrete interferon-gamma (IFN-*γ*), tumor necrosis factor alpha (TNF-*α*), and lymphotoxin to corrupt liver grafts via delayed-type hypersensitivity (DTH), in the process of which macrophages may be a primary participant and play a critical role. (b) Immune rejection mediated by humoral immunity. B cells can directly recognize donor antigens without the antigen presentation of APC, which will be subsequently activated and differentiated into plasmocytes with the help of Th2 cells, and secrete antibodies to destroy donor cells. TCR: T cell receptor; BCR: B cell receptor; HLA: human leukocyte antigen.

**Figure 2:**
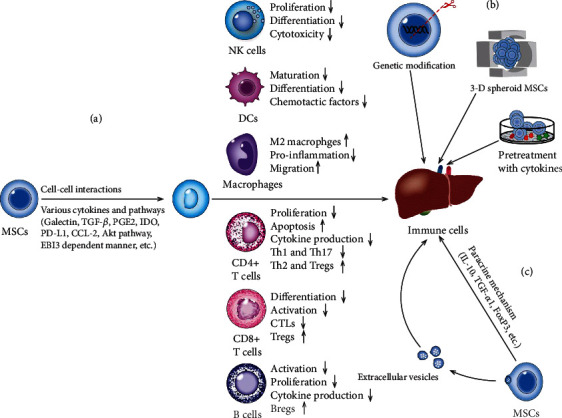
The mechanism of immune tolerance induced by mesenchymal stem cells (MSCs). (a) MSCs regulate the maturation, proliferation, differentiation, and apoptosis of immune cells to generate a tolerogenic microenvironment via cell-cell interactions and diverse cytokines and signal pathways. (b) Preconditionings to induce immune tolerance. The capacity of immune regulation of MSCs might be influenced by the pretreatment with cytokines, genetic modification, and culture method (three-dimensional (3D) culture or 2D culture). (c) Paracrine mechanisms and extracellular vesicles derived from MSCs to induce immune tolerance after liver transplantation. TGF-*β*: transforming growth factor-beta; PGE2: prostaglandin E2; IDO: indoleamine 2,3-dioxygenase; PD-L1: programmed cell death-ligand 1; CCL-2: chemokine ligand-2; EBI3: Epstein-Barr virus-induced 3; IL-10: interlukin-10; FoxP3: forkhead box P3; NK: natural killer; DCs: dendritic cells; CTLs: cytotoxic T lymphocytes; Tregs: regulatory T cells; Bregs: regulatory B cells; Th1: T helper 1.

Rejection is very common in liver transplant patients. If the rejection is severe or recurring, it may affect the long-term survival of the patient. Acute rejection after liver transplantation usually requires high-dose immunosuppressive therapy, which has serious and toxic side effects (Figure 1). Therefore, it is imperative to find a safe and effective method to prevent rejection in liver transplantation patients. MSC transplantation is considered to be a promising strategy to induce immune tolerance based on the unique immunomodulatory properties of MSCs in the interactions with the surrounding host microenvironment, in which MSCs can regulate the function of immune cells through cell-cell interactions and secretion of various cytokines. Moreover, pretreated with cytokines, genetically modified, or 3D cultured, MSCs may regulate the immune system more effectively and thus more obviously improve the survive rate and prognosis in patients with liver transplantation (Figure 2).

### 2.3. Regulation of Immune Cells in Response to Liver Transplantation

The liver is an immune tolerant organ, of which multiple cells express low levels of MHC antigens and it is difficult to induce innate or adaptive immune responses in the liver [47]. Various immune cells, including natural killer (NK) cells, dendritic cells (DCs), T cells, B cells, Kupffer cells (KCs), and sinusoidal endothelial cells (LSECs), are located in liver tissues, most of which migrate from the peripheral blood and trigger immune responses to invading pathogens in an antigen-specific way. Moreover, past research has revealed that intrahepatic immune cells exerted a high degree of immunosuppressive effect through cell-cell interaction and secretion of cytokines after liver transplantation [48].

There will be two sources of immune cells in the blood circulation of the recipient, donor liver-resident cells and the recipient immune cells, responding to the changed microenvironment after liver transplantation. The former are graft-derived immune cells that enter the peripheral blood of the recipient, and the latter are immune cells from the recipient that invade the liver transplant [49–51]. Transplanted organs are recognized by the recipient's immune system as “alien components,” which are attacked, destroyed, and ultimately eliminated by the recipient's immune system. Dendritic cells and macrophages uptake MHC molecules on donor tissue cells and present antigen information to B cells and T cells. And T cells, as the main participants in cellular immunity, also affect humoral immunity and innate immunity by secreting various cytokines [52]. A prospective study by Kim et al. [53] showed that calcineurin inhibitor-based immunosuppression protocols decreased the percentage of regulatory T cells (Tregs) in peripheral blood of living donor liver transplantation recipients and the percentages of effector T helper 17 (Th17) cells and memory B cells are unaffected, which indicated that cellular immunity might play a leading role in the rejection of solid organ grafts.

Notably, natural killer cells occupy an important position in the human immune system, not only related to antitumor and antiviral infection and immune regulation but also involved in the occurrence of hypersensitivity and autoimmune diseases [54]. As the most common cytotoxic innate lymphocytes, NK cells account for up to 30% of the intrahepatic lymphocyte population, which directly interact with T cells and DCs. An observation by Garcia de la Garza et al. [55] reported that 24 liver transplantation patients with immunosuppressive side effects underwent progressive immunosuppressive withdrawal therapy, 15 of which developed immune tolerance and 9 developed immune rejection. The secretion of cytokines such as tumor necrosis factor alpha (TNF-*α*), IL-2, IL-4, IL-6, and IL-10 was lower in tolerant recipients than in nontolerant recipients. On the contrary, the percentage and absolute counts of NK cells in the peripheral blood of recipients with immune tolerance are higher than those of recipients with immune rejection, which indicated the great potential value of NKs in inducing immune tolerance. Similar findings by Jamil et al. [56] reported that NK cells in non-HCV liver transplantation recipients were hypofunctional with weakened cytotoxicity, decreased expression of NKp30 and NKp46, and reduced secretion of interferon-gamma (IFN-*γ*), which was generated via the IL12/STAT-4 signaling pathway. Consequently, STAT4 might be a potential therapeutic target for inducing NK cell tolerance to the transplanted liver. Besides, NKG2D, a class of receptor mainly expressed on the surface of NK cells, may also play a key axis role to induce immunological tolerance in liver transplantation [57].

In addition to NK cells, liver sinusoidal endothelial cells (LSECs) also exert a strong impact on the immune rejection and tolerance of liver allotransplantation [58]. LSECs participate in the construction of the hepatic sinusoid wall and act as a control hub for regulating and supervising the transport of molecules and cells between liver parenchyma and peripheral blood. As the largest number of nonparenchymal cells in the liver, LESCs are responsible for plasma ultrafiltration, scavenger function, and regulation of liver microcirculation and immunity, based on which they are increasingly recognized as important participants in liver immunity [59]. It is well known that myeloid antigen-presenting cells (APC) cross-present foreign antigens to CD8+ T cells on major histocompatibility class I molecules (MHC-I), thereby producing a proinflammatory response to combat microbial infections. In contrast, Limmer et al. [60] reported that LSECs use the same cross-presentation approach to feedback the information of soluble exogenous antigens to CD8+ T cells via MHC-I, creating a microenvironment for immune tolerance after liver transplantation. Besides, LESCs that endocytose allogeneic cells can attenuate the proliferative activity of CD4+ T cells, inhibit their secretion activity of IL-2, and thereby induce the immune tolerance of CD4+ T cells to allogeneic transplanted organs, at least partially via the Fas/FasL pathway [61]. Moreover, a study published in *Hepatology* by Kruse et al. [62] has first proven that LESCs having expression of CD25 (low) and lacking expression of forkhead box protein 3 (FoxP3) might be the expanding group of regulatory T cells. In this article, LSECs could prime CD4+ T cells to be suppressive phenotypes lacking marker cytokine production for effector cells, which makes effector cells maintain their stability even reencountering with immunogenic antigens in vivo. As to other types of intrahepatic innate immune cells, it was reported that Kupffer cells (KCs) could stimulate T cells to wither through the Fas/FasL pathway [63], promote the proliferation of Tregs to inhibit the immunoreaction of cytotoxic T lymphocytes (CTLs) [64], decrease hepatocyte apoptosis, and attenuate the liver injury in recipients [26], and those with high expression of programmed cell death-ligand 1 (PD-L1) could reduce the proliferation and functions of T cells via direct cell-cell contact to prolong liver allograft survival [65]. Nakano et al. [66] showed that liver-derived DCs significantly inhibit the immune response of T cells and promote the activation of Tregs to generate a tolerance via secretion of IL-10; besides, upregulation of TGF-*β*1 and downregulation of IL-12 were also reported to participate in inducing transplantation tolerance.

In conclusion, intrahepatic immune cells, especially innate immune cells, play a pivotal role in maintaining the balance between inflammation and anti-inflammation for the short- and long-term outcomes after liver transplantations. Modification of immune cells through immunosuppressive drugs, cytokines, MSCs, or other means may reduce the rejection rate of liver grafts and prevent rejection and graft loss in liver transplant recipients.

### 2.4. The Interaction between MSCs and Immune Cells

Mesenchymal stem cells establish a stable and balanced microenvironment by regulating innate and adaptive immune cells, and the interaction between MSCs and the immune system modulates inflammation in vivo and in vitro [67–71]. The cell-cell interaction and paracrine between MSCs and immune cells are reported, the guarantee for successful treatment of immune-related diseases. Relevant in vitro experiments have shown that MSCs have powerful immune regulation ability that could affect almost all immune cells, such as macrophages, DCs, NK cells, T cells, and B cells to generate a tolerogenic microenvironment.

### 2.5. The Interaction between MSCs and Innate Immune Cells

For NK cells, Li et al. [67] showed that MSCs could interfere with NK cells on the cell cycle, by arresting NK cells in the G0/G1 phase, diminishing the ratio of the S and the G2/M phase, and inducing apoptosis in a dose-dependent manner. Two interesting phenomena, decrease in CD69 expression on the NK surface and increased ratio of CD4 (+) CD25 (+) CD127 (low) T regulatory cells, were observed in the coculture system of NK cells, and MSCs may contribute to this process. Fan et al. [72] demonstrated that human fetal liver MSC-derived exosomes inhibited NK proliferation, differentiation, and cytotoxicity via TGF-*β*. Moreover, MSCs cannot only effectively inhibit IL-2-induced NK cell proliferation but also prevent cytotoxic activity and cytokine production. Notably, indoleamine 2,3-dioxygenase (IDO) and prostaglandin E2 (PGE2) were reported to be the key mediators in MSC-induced inhibition of NK cells [73, 74].

With respect to DCs, MSCs induce DC immune tolerance via paracrine hepatocyte growth factor (HGF), and the mechanism is reported to be associated with the activation of the Akt pathway [75]. Similar experimental results were reported in another study by Lu et al. [76]. In this article, decreased levels of MHCII, CD86, and CD40 and increased levels of PD-L1 were observed in regulatory DCs cocultured with MSCs, the process of which was realized by the activation of Notch signaling. Dendritic cells, the most effective antigen-presenting cell in the body, play a critical role in efficient cross-talk with different cells of the innate immunity and initiate immune responses by promoting antigen-specific T cell activation [68]. MSCs interfere with the differentiation of DCs to maintain them in the immature stage and suppress the proinflammatory factors released by DCs [77]. In detail, a variety of secreted factors, such as PGE2, TNF-*α*, and Foxp3, as well as cell-cell contact were reported to be involved in this process. Regarding macrophages, TGF-*β* secreted by MSCs can polarize macrophages towards the anti-inflammatory phenotype (M2), attenuate the proinflammatory cytokine levels, and increase phagocytosis through the Akt/FoxO1 pathway, providing potential treatment modalities for sepsis [78]. An experimental analysis by Liu et al. [79] suggested that MSCs significantly upregulate the recruitment of macrophages to mitigate the pathology of colitis and promote the repair of tissue damage.

### 2.6. The Interaction between MSCs and Adaptive Immune Cells

In the clinical application of organ transplantation, suppressing the function of the adaptive system (humoral immunity and cellular immunity) efficiently is considered to be a crucial point among the immunomodulatory properties of MSCs [68]. For B cells, previous studies suggested that MSCs inhibited B lymphocyte-related humoral immune responses by blocking B cell activation, proliferation, and cytokine production, which is achieved most likely through the direct contact between MSCs and T cells, trapping B cells in the G0/G1 phase of the cell cycle [80, 81]. Cho et al. [69] indicated that MSCs promoted the conversion of B cells to an anti-inflammatory phenotype, namely, regulatory B cells (Bregs), to ameliorate immune responses in an Epstein-Barr virus-induced 3- (EBI3-) dependent manner. Moreover, the PI3K-AKT signaling pathway and actin skeleton regulation have been proven to be the key mediators of MSC/B cell communication, which was identified and functionally validated in vitro experiments by Adamo et al. [82].

Regarding T cells, previous studies demonstrated that MSCs suppressed cellular immune response by inhibiting the proliferation and activation of T cells, promote apoptosis, and regulate the proportion of subsets, which manifests as increased Tregs and Th2 cells, and decreased T helper 1 (Th1) and Th17 cells [70, 83]. This immunomodulatory capacity of MSCs may be related to their function of participating in tissue repair in vivo where transient and local suppression of immune response will be beneficial to differentiation. Regarding the mechanism by which MSCs affect T cell activity and phenotype, some authors believe that it is closely related to the functional status of DCs [84]. Kim et al. [71] conducted an in vitro experiment that only CD4+ T cells (with phytohemagglutinin stimulation) and MSCs are cocultured, and MSCs still have an immunosuppressive effect on the activation and proliferation of T cells. This experiment directly verifies that the role of cell-cell contact among the immunosuppressive properties of MSCs needs more attention. In order to further explore whether there was a special channel underlying cell-cell contact, Ma et al. [85] revealed that knockdown of PD-L2 makes the number of Th17 cells increased when cocultured with MSCs.

In addition to direct cell-cell contact, soluble molecules are also thought to mediate T cell suppression, because MSCs can still inhibit T cell proliferation even without cell-cell contact. Khosravi et al. [86] investigated the role of cell-cell contact and cytokine secretion by BM-MSCs on the stability, induction, and suppressive functions of Tregs using flow cytometry, ELISA, real-time PCR (RT-PCR), and high-resolution melting technique. Subsequently, they found that ubiquitination might be a potential mechanism by which MSCs converted conventional T cells to suppressive and stable Tregs. Moreover, many other molecules known to be possibly involved in MSCs include TGF-*β*, adenosine signaling, hepatocyte growth factor (HGF), IDO, PGE2, insulin-like growth factor/insulin-like growth factor-binding protein-4, heme oxygenase-1 (HO-1), human tissue phase capacitive antigen-G5 (HLA-G5), chemokine ligand-2 (CCL2), IL-10, galectin-1, and galectin-3 [84, 87, 88], all of which have been reported to be involved in the suppression of T cell proliferation and phenotypic transition. To elucidate the potential mechanism of interaction between cytokines secreted by MSCs and T cells, Kim et al. [71] knocked down FoxP3 expression on the surface of purified T cells, cocultured them with MSCs for 3 days, and finally count the proportion of Tregs in the entire culture system. The results suggested that downregulation of FoxP3 significantly attenuated the ability of MSCs to promote the differentiation of conventional T cells into Tregs. Further, the bridging component between FoxP3 and MSCs was confirmed to be a series of cytokines, including anti- and proinflammatory cytokines (IL-2, IL-6, IFN-*γ*, IL-4, IL-5, IL-13, etc.) and members of the TGF-*β* family secreted from MSCs. However, the molecular pathways by which MSCs inhibit the proliferation and differentiation of T cells are not yet fully understood, and this is the focus of research in the future. As the clinical application of MSC transplantation becomes more popular, the mechanism governing cytokine interactions between MSCs and T cells will certainly come to be the focus of research in the future.

### 2.7. The Paracrine of MSCs in Protecting Recipients of Liver Transplantation

In addition to interactions with liver immune cells to achieve immunosuppression or transplant tolerance, MSC transplantation can also protect liver transplant recipients from acute or chronic rejection-induced damage via paracrine mechanisms. An animal study by Niu et al. [89] showed that MSC transplantation could prolong the survive rate of liver transplantation recipients by upregulating the expression of TGF-*β*1, FoxP3, IL-10, and cytotoxic T lymphocyte-associated antigen-4 (CTLA-4) as compared to MSC-untreated recipients; the suppressive capacity and expression level of FoxP3, IL-10, and CTLA-4 in CD4+CD25+ regulatory T cells in the MSC administration group are significantly higher. Coincidently, Gao et al. [90] performed liver transplantation followed by autologous MSCs delivered into the portal vein system in rats and found that MSCs could reduce inflammatory responses, attenuate acute rejection, and thus enhance liver regeneration; in this experiment, MSCs markedly promoted the expression of PCNA, TGF-*β*1, and IL-10 in the allograft, whereas those of IL-2 and IL-17 expression levels were significantly reduced. Besides, other cytokines, such as TGF-*α*1, PGE2, IL-6, IL-23, and IFN-*γ*, were also reported to be involved in the acute rejection and transplantation tolerance regulated by MSCs [43, 91, 92]. Moreover, Chen et al. [46] found that MSCs could significantly attenuate the rejection rate of allogeneic liver transplantation and prolonged the median survival time of liver transplantation recipients, which might be achieved via upregulation of PD-L1 expression by downregulating the expression of miR-17-5p. To further explore the action of molecules secreted by MSCs in the liver transplantation, Du et al. [93] systemically infuse the MSC-conditioned medium in the recipients and showed that it could prevent the cell death of hepatocytes and LSECs and promoted their regeneration via reducing neutrophil infiltration, KC activation, and secretion of inflammatory factors while upregulating the levels of matrix metallopeptidase 9 and vascular endothelial growth factor (VEGF) in the liver grafts.

Even more to the point, immunomodulatory paracrine of MSC is tightly regulated by extrinsic microenvironment and intrinsic molecules such as the telomerase-associated protein RAP1/NF-*κ*B signaling pathway. RAP1 is a well-known telomeric repeat-binding factor 2-interacting protein 1 (Terf2IP), acting as a novel modulator involved in the nuclear factor kappa B (NF-*κ*B). RAP1 could negatively regulate telomere length in human MSCs, and RAP1 deficiency enhanced the self-renewal of MSCs and delayed steam cell senesces [94]. In an animal model study concerning myocardial infarction (MI), Zhang et al. revealed that knockdown of Rap1 resulted in downregulation of NF-*κ*B activity characterized by reduced proinflammatory paracrine cytokines (TNF-*α*, IL-6, and monocyte chemotactic protein-1) and increased the resistance to hypoxia-induced apoptosis in MSCs. Further, in an in vivo research phase, Zhang et al. also found that transplantation of Rap1^−/−^-MSCs significantly improved heart function, prevented cardiomyocyte apoptosis, decreased infarct size, and inhibited inflammation compared with the MSC group [95]. In contrast, a recent study by Ding et al. displayed an opposite finding that Rap1 was an essential target to maintain the normal immunomodulatory ability of MSCs, in which Rap1 deficiency provoked paracrine dysfunction of MSCs and impaired their responsive immunosuppression in allograft rejection of heart transplantation [96]. Activated Rap1 could improve the survival, adhesion, and differentiation of transplanted MSCs to restore myocardial function in the infarct area [97]. Therefore, the role of Rap1 in the function of MSCs still needs to be elucidated in the further research, especially in the field of liver transplantation. Moreover, it has been reported that there are active interactions between inflammatory environment and mitochondrial transfer of MSC involved in MSC immunomodulation and tissue repair including liver regeneration. For example, the proinflammatory cytokine CXCR3 has been reported to induce mitochondrial dysfunction of hepatocytes, which contributes to the pathogenesis and development of nonalcoholic steatohepatitis (NASH) [98]. In turn, such proinflammatory environment also promoted high efficiency of mitochondrial donation from transplanted MSCs to repair mitochondrial damaged tissues [99–101] and enhance macrophage phagocytosis [102], thereby improving the survival of grafts and long-term prognosis. However, as of now, no matter whether it is RAP1 or mitochondrial transfer, there is almost no data about their application in liver transplantation and it is urgently needed to fill this gap in the near future.

Besides paracrine signaling, extracellular vesicles (EVs), including microvesicles and exosomes, have been identified as a newly discovered mediator in the intercellular communication system. Notably, MSC-derived extracellular vesicles (MSC-EVs) have been shown to possess the comparable therapeutic capabilities as MSCs themselves. In a variety of models in vivo, it has been observed that they can inhibit the inflammatory response and reduce oxidative stress and fibrosis. By converting the proinflammatory immune response into a tolerant immune response, MSC-EVs might create an immune tolerance microenvironment that allows endogenous stem cells and progenitor cells to obtain immune exemption and then gradually repair affected tissues and promote regeneration [103]. It was reported that noncoding RNAs within MSC-EVs may play a regulatory role in the regulation of matrix remodeling, epithelial-mesenchymal transition, inflammation resolve, and tissue regeneration [104]. More recently, a study published in *Front Genet* showed similar results that MSCs reduced liver inflammatory in acute-on-chronic liver failure (ACLF) via the regulation of the miRNA content within exosomes in the hepatic microenvironment. It was observed that miR-20a-5p was downregulated in hepatocytes and exosomes, while the expression of chemokine C-X-C motif chemokine ligand 8 (CXCL8) was upregulated in hepatocytes [105]. On the other hand, Rong et al. [106] reported that MSC-derived exosomes could effectively ameliorate liver fibrosis, including a reduction in collagen accumulation, inhibition of inflammation, and increased regeneration of hepatocytes through the Wnt/*β*-catenin pathway. And a newly published study from the United States put out a novel antifibrotic approach directly targeting signal transducer and activator of transcription 3 (STAT3), which was an important transcription factor associated with liver fibrosis, and this process was accomplished based on the administration of MSCs-derived exosomes embedded with small interference RNAs and antisense oligonucleotides [107]. As for whether MSC-EVs can improve the prognosis of liver transplantation patients, most of the recent studies focused on the process of ischemia-reperfusion injury (IRI) after surgery [108–110]. It was reported that the administration of MSC-EVs ameliorated IRI perhaps by enhancing autophagy [108], increasing cell viability and suppressing nuclear factor kappa B activity in hepatocytes [109], or reducing CD154 expression on CD4+ T cells via chaperonin containing TCP1 subunit 2 (CCT2) [110]. Although the exact mechanism of MSC-EVs to improve the prognosis of liver transplantation has yet to be fully seen, MSC-EVs are worth recognizing as an emerging and very promising therapeutic agent, especially in the regulation of immune rejection.

### 2.8. Modification of MSCs to Improve the Therapeutic Effect in Liver Transplantation

The immunomodulatory properties of MSCs in liver transplantation have been recognized, and investigations designed to increase the therapeutic potential of MSCs have also been carried out intensively. It is remarkable that cytokine pretreatment, genetic modification, or three-dimensional (3D) culture can enhance the immune regulation ability of MSCs to regulate immune cells in vitro and in vivo, which may be an effective method to improve the immune regulation ability of MSCs in LT. Hong et al. [111] demonstrated that MSCs pretreated with IFN-*γ* can upregulate the expression of PDL-1, CD54, MHC-I, and MHC-II, boost the immune suppression capacity in the vivo, improve the homing ability to liver tissue, and attenuate the acute liver transplantation rejection in rats. Moreover, transplantation of TGF-*β*-overexpressing MSCs was reported to be more likely to induce a local immunosuppression in liver grafts after transplantation, alleviate the acute immune rejection, and thus reduce the mortality of liver transplantation rats [112]. A similar animal model constructed by Niu et al. [113] indicated that MSCs genetically engineered with interleukin-10 (IL-10) could significantly prolong the mean survival time of rats, in the process of which the expression of cytokines (IL-17, IL-23, IL-6, IFN-*γ*, and TNF-*α*) decreased significantly compared with the placebo group, whereas those of FoxP3, IL-10, and TGF-*β*1 increased in a time-dependent manner. Importantly, some recent studies have shown that heme oxygenase-1 (HO-1) may be a key gene that enhances the immunomodulatory effects of MSCs. HO-1-overexpressing MSCs can significantly decrease the rejection activity index and increase the median survival time of the allograft liver via the downregulation of proinflammatory cytokines and activity of NK cells and upregulation of the Tregs and anti-inflammatory factors [114]. Of note, it was reported that HO-1-transduced MSCs exert protective effects on liver grafts against acute rejection injury, possibly via upregulating the expression of autophagy-related proteins through the ERK/mTOR signaling pathway [115]. Besides, HO-1-overexpressing MSCs promoted the regeneration of liver grafts by improving the activity of mitochondrial aspartate aminotransferase, ameliorating the microcirculation of hepatic sinusoids, and recovering the energy metabolism of impaired hepatocytes [116].

Notably, in traditional two-dimensional (2D) culture, cell adsorption only occurs on the side in contact with the culture surface, many of which will become flatter, divide abnormally, and may lose their differentiated phenotype in the process of cultivation. In the 3D culture, cell adsorption can occur on the entire cell surface. The degree of cell adsorption and stretching affects its proliferation, apoptosis, and differentiation, and thus, the 3D culture can establish physiological cell-cell and cell-extracellular matrix interactions, simulating the specificity of natural tissues. Early in 2012, Ji et al. [117] compared the cell proliferation and hepatic differentiation of MSCs in a liver biomatrix scaffold with those in a two-dimensional substrate. Consequently, hepatic differentiation of MSCs by the 3D culture exhibited extensive functions and indicated that decellularized liver matrix bioscaffolds might be a promising tool in the liver tissue engineering. To investigate action mechanisms and therapeutic effects of 3D spheroid MSCs, Zhang et al. [118] isolated human adipose-derived MSCs, cultured them as 3D spheroids, and transplanted them into liver fibrosis model mice. In the result, 3D spheroid MSCs exerted more conducive effects in ameliorating liver fibrosis than 2D cultured cells, possibly via upregulating the expression of antifibrotic factors, such as hepatocyte growth factor (HGF), IL-6, and insulin growth factor 1 (IGF-1). Similar findings were reported by a very recent study conducted by Choi et al. [119], in which 3D-cultured MSCs exhibited robust antifibrotic potential based on the high levels of HGF, IGF-1, and stromal cell-derived factor- (SDF-) 1 genes, and high numbers of hepatocytes expressing Ki-67 were also detected in the MSC-3D-injected livers. Simultaneously, 3D culture of MSCs also plays a pivotal role in the improvement of IRI. MSCs delivered in a three-dimensional aggregate form could make up the shortfalls of the therapeutic effects of MSCs limited by the low engraftment of stem cells [120]. More importantly, delivering MSCs in the 3D aggregate form could boost effects of MSCs in restoring structure and function after renal IRI, and MSCs in 3D aggregates were less susceptible to oxidative and hypoxia stress and secreted more proangiogenic factors compared with MSCs cultured in the 2D monolayer. Another study concerning liver IRI by Sun et al. [121] showed that 3D MSC transplantation had a better therapeutic effect compared with 2D MSCs in treating hepatic IRI in rats. In serological testing, 3D MSCs reduced the secretion of proinflammatory chemokines while increasing the production of VEGF, the mechanism which is the role of zinc finger CCCH-type-containing 12A (ZC3H12A), an important anti-inflammatory gene whose product could destabilize and even turnover the mRNA of proinflammatory genes (IL-1, IL-2, IL-6, CXCL1, CXCL2, etc.) at the posttranscript level, significantly upregulating in 3D MSCs.

Based on the above analysis, modification of MSCs, such as preconditioning with cytokines, genetic modification, and 3D culture, has shown great potential in the fields of tissue engineering. But so far, most studies are still limited to in vitro experiments or animal models, and the modification of MSCs for tissue repair after liver transplantation is also limited to plates concerning liver fibrosis and IRI. More various modification methods, more precise targets, and deeper molecular and even gene regulation mechanisms will inevitably become the research hotspots of MSCs inducing immune tolerance after liver transplantation.

## 3. Conclusion

According to available information from various investigations (in vitro, animal model, preclinical and clinical studies, etc.), MSC administration has been considered to be a feasible and safe strategy in improving the short- and long-term prognosis of liver transplantation. However, the application of MSCs needs to be cautious in certain types of patients, especially those with hepatocellular carcinoma, because the role of MSCs in cancer progression remains controversial. The application of MSCs following liver transplantation could promote immune tolerance in liver grafts, but whether MSCs could perfectly substitute an immunosuppressant is still a question.

MSCs have been proven to regulate the immune system effectively to inhibit acute rejection, promote regeneration of damaged liver tissue, and induce the immune tolerance microenvironment in liver transplant patients. Growing evidence indicates that soluble molecules, cell-cell contact, paracrine signaling, and extracellular vesicles have been identified as mediators in the intercellular communication system whereby MSCs exert their immunosuppressive effects. Although various cytokines, manners, and signal pathways have been confirmed to play a pivotal role in the interaction between MSCs and the surrounding host microenvironment to induce a tolerogenic microenvironment, the data are mostly obtained from in vitro experiments and animal models; thus, the exact mechanisms of immune regulation of MSCs, especially in vivo, need to be further explored. It is worth highlighting that EVs have shown promising therapeutic prospects currently, but whether EVs can replace cell therapy in liver transplantation remains to be confirmed. Promoting the immunomodulatory capacity and homing ability of MSCs through phenotypic modification via pretreatment with cytokines, genetic modification, or 3D cultures has been carried out and has acquired encouraging results. Nevertheless, whether the genetic modification of MSCs increases the risk of malignant tumors in recipients is still controversial, and further research is needed.

In summary, MSC-based therapy is a promising tool in regenerative medicine for inducing immune tolerance after liver transplantation, but the exact molecule mechanisms and the problems that may be encountered in clinical practice still need large-scale and longer-term clinical trials to support.
